# On the Resection of Both Upper Jaw Bones
*[We have received this very interesting essay on an important surgical operation direct from the author, with the following letter, which we make no apology for laying before our readers. We feel highly flattered at his selection of our Journal for its communication to the profession in the British islands; and in revising it for the press, we have altered the orthography as slightly as possible, being anxious to retain Dr. Heyfelder's excellent, though, of course, somewhat idiomatic English translation.—Ed."The accompanying manuscript I have the honor to send, with the request that you will, after some inspection and correction, insert it in your much read and highly valued Journal. I found in No. xxxi, page 36, for the year 1853, a notice, by Butcher, of the resection of both superior maxillary bones performed by my father; and I think I send you a not wholly valueless dissertation on this subject, whereby it is my object, at the same time, to prove the fact, to furnish a useful contribution to scientific surgery, and to extend my father's fame. But, at all events, I should also consider myself extremely fortunate if this should lead to a further scientific intercourse, which might keep me in communication with you.—6, *Promenade-place, Munich, October* 5, 1856.]


**Published:** 1857-04

**Authors:** Oscar Heyfelder

**Affiliations:** Private Lecturer on Surgery and Ophthalmic Medicine in the Unversity of Munich; Member of the Academy of Naturalists at Breslau, and the Academy of Surgery at Madrid; of the Medical Societies at Vienna, Paris, Leipzig, Munich, Strasburgh, Brussels, Erlangen, Valencia; of Legal Medicine in Baden; and of the Surgical and Anatomical Society at Paris.


### SELECTED ARTICLES.

### ARTICLE VI.

*On the Resection of both Upper Jaw Bones.** By Dr. Oscar Heyfelder, Private Lecturer on Surgery and Ophthalmic Medicine in the University of Munich; Member of the Academy of Naturalists at Breslau, and the Academy of Surgery at Madrid; of the Medical Societies at Vienna, Paris, Leipzig, Munich, Strasburgh, Brussels, Erlangen, Valencia; of Legal Medicine in Baden; and of the Surgical and Anatomical Society at Paris.

It is well known that in the year 1824, Roger, (according to Velpeau,†) and soon afterwards Liston‡ and Dupuytren,§ performed, to a greater or less extent, partial resections of both upper jaw bones. The first total removal of these bones must,

* [We have received this very interesting essay on an important surgical operation direct from the author, with the following letter, which we make no apology for laying before our readers. We feel highly flattered at his selection of our Journal for its communication to the profession in the British islands; and in revising it for the press, we have altered the orthography as slightly as possible, being anxious to retain Dr. Heyfelder's excellent, though, of course, somewhat idiomatic English translation.—Ed.

"The accompanying manuscript I have the honor to send, with the request that you will, after some inspection and correction, insert it in your much read and highly valued Journal. I found in No. xxxi, page 36, for the year 1853, a notice, by Butcher, of the resection of both superior maxillary bones performed by my father; and I think I send you a not wholly valueless dissertation on this subject, whereby it is my object, at the same time, to prove the fact, to furnish a useful contribution to scientific surgery, and to extend my father's fame. But, at all events, I should also consider myself extremely fortunate if this should lead to a further scientific intercourse, which might keep me in communication with you.—6, *Promenade-place, Munich, October* 5, 1856.]

† Méd. Opérat., t. ii. p 628.

‡ Lancet, November, 1836, p. 267.

§ Leçons Orales d. Cl. Chir., 2ème edit., t. ii. p. 453.

however, be ascribed to J. F. Heyfelder,* who accomplished it in the year 1844, and repeated it twice in the years 1850 and 1852.

In two cases, I aided in the operation as medical assistant of the hospital; in all three cases I had occasions to see the patient previous and subsequent to the operation, and to study the diseased parts removed by the resection.

Case I.—Andreas Schmidt, aged 23, entered the surgical Clinique at Erlangen, on the 13th of June, 1844, in consequence of a tumor, which, according to the assertion of the patient, had issued a year previously from the hind part of the palate, and had extended itself over both upper jaw bones. By this pseudoplasma, which increased every day, the nose was forced upwards, and at the same time pressed flat; the palate was dislodged and lowered upon the tongue; the face swollen, and resembling that of a monkey; both the breathing and speech impeded; sleep restless, and troubled with anxious dreams. Except the two middle incisors, all the teeth were preserved loose, but in a healthy state. The tumor itself was in all its parts hard, uneven, not very sensible to the touch, and not extending beyond the limits of the jaw bones. No signs of an existing dyscrasia, nor of earlier diseases. Only latterly the face, and especially the region of the upper jaw, were affected from time to time with severe pains. From these antecedents, as Professor Heyfelder observed in presenting the patient to his class, he was authorized to conclude that the tumor was not of a benign nature; on the contrary, all seemed to prove it was a medullary carcinoma, which required not only a mere excision, but even the removal of the base of the evil. But this could not be effected except by the resection of both ossa maxillaria. This operation not having hitherto been performed, Professor Heyfelder could not conceal from himself either the difficulty or the danger connected with so important a surgical proceeding. On the contrary, he had to consider, that in case of

*J. F. Heyfelder was then Professor of Surgery at Erlangen, afterwards Chief Surgeon of the Finland Army; now employed at St. Petersburgh, in the Medical Department of the Ministry of War.

hemorrhage, all means were in his hands to staunch it, and that the pain necessarily connected with such deep incisions would not be more violent than in various other operations. But it seemed obvious, that with the end of the operation, all danger would not cease; and that he had to fear a subsequent bleeding, or, more than that, an intense inflammation of all the adjacent parts, even of the brain and its membranes; but this being of a traumatic origin, it might be prevented or removed by suitable means.

As he had to remove a disease which, if left to itself, might extend more and more, and even seriously endanger life, the deformity produced by this operation could scarcely be taken into consideration, so much the rather as the tumor itself actually produced a high degree of deformity, which would at all events, be diminished by its removal.

Therefore, the danger and consequences of the operation were counterbalanced by the probability of a favorable result, and Professor Heyfelder, consequently performed the total resection of the bones, in the presence of Professor Canstadt, Professor V. Siebold, Professor Ried, Professor Winterich, in the following manner:

The patient was seated in an arm chair; his head supported against the breast of an assistant, and fixed in this situation. The operator made two incisions from the outer angles of the eyes to the corners of the mouth, and pared all the softer parts away from the tumor to the inner angles of the eyes and the nasal bones. The flap thus formed being drawn upwards over the forehead, and the orbital margin being cleared down to the inferior ridge, he separated on both sides the juncture between the jaw and cheek bone by means of a chain-saw introduced through the inferior orbital fissure. In the same way, the separation of this bone with the nasal bone was effected. Then the vomer and the other junctions still existing were divided by a bone forceps, and the soft palate separated from the hind edge of the hard palate. After that, pressure with a chisel upon the upper part of the tumor sufficed to dislocate the upper jaw bones out of the principal junctions, and so to finish the operation. It lasted three-quarters of an hour, as three times fainting of the patient had necessitated rather long interruptions.

The loss of blood had not been extremely great, as torsion and compression sufficed to stop the bleeding during and after the operation.

We had before us a hollow, caused by the removal of the natural partition wall between the mouth and nose, producing a very disagreeable impression.

The two long wounds of the cheeks were united with twenty-six insect needles, and cold fomentations were applied. The man operated on was nourished during the first day by means of a syringe, with which he received broth and cooling beverages; the second day by means of a spoon; the third and fourth days he took fluids out of a cup. No extraordinary reaction appeared.

On the removal of the dressings on the fourth day, the cheek wounds were found almost in all parts united; only in some places a superficial suppuration had taken place.

The thirteenth day after the operation, Schmidt was presented to the Physico-Medical Society at Erlangen; on the thirty-second day after that, he was removed home. No external deformity was to be seen, the two incisions having healed perfectly throughout. In the interior of the mouth could be observed a fissure thirteen lines long and three lines broad; the removed parts were replaced by a tissue, which seemed hard on the surface, but somewhat œdematous towards the fissure. The soft palate and uvula were, contrary to all expectations, in their natural position, and of a normal condition; the process of eating and drinking was unaffected; the speech was better, i. e. more intelligible, than before the operation; the nose had a more natural form, and was no longer as it had been heretofore, pulled upwards and pressed flat; respiration free, and sleep sound.

The pseudo-plasma, into which both upper jaw bones were changed, appeared as a dense, hard, bacon-like tumor, rich in blood, in which, on microscopical examination, it was seen the cellular formation prevailed. Both jaw bones were preserved in their contiguity, and in this form, though enormously enlarged, the preparation is kept in the anatomical collection of the Surgical Clinique.

Half a year after the operation the man was perfectly well, so that he even could work in the fields. But seven months later we received the information that a new pseudo-plasma was developed on his forehead, with excruciating pains; it increased rapidly, and occasioned his death in the month of October, 1845, fifteen months after the operation.

Case II.—George Lochner, aged 55, who never before had been seriously sick, was affected with cancer twelve years previously, on the right side of the upper lip, which increased slowly, and which was removed by the knife three years afterwards. During two years the man remained free of a relapse; but at the end of this period a wart-like little tumor arose near the cicatrix, which soon changed, with tormenting pain, into an open ulcer, which extended itself over the right side of the upper lip, over the nose, and over the palate.

When the patient entered the Surgical Clinique on the 21st of January, 1850, his estate was as follows:

A disgusting carcinomatous ulcer extended itself from the right corner of the mouth to over the greater part of the upper lip, and had already destroyed the right wing of the nose. In the same way the cartilaginous part of the lower division of the nose became attacked with the disease, so that the greater part of the upper lip, and more than half the nose, presented an ulcerating surface, covered with fungosities, easily bleeding, and painful at the slightest touch.

The whole of the palatal arch was degenerated into a hilly, cancerous tumor, which extended itself equally over both upper jaw bones, and to the insertion of the soft palate.

Some remains of the teeth stuck in the alveoli, and augmented the digusting impression produced by the view of this patient; the more so as a suppuration, of a cadaverous odor, covered the ulcerating surface, and the patient showed the symptoms of a lengthened suffering, the body having wasted away, the complexion of his face being clay-colored, and the features disfigured.

In order to save the man's life, the removal of the cancer was necessary; it had attained such a circumference, that the total resection of both upper jaw bones, the excision of the greater part of the upper lip, and of all the cancerous parts, soft and hard, of the nose, seemed unavoidable—an operation that could not be done without deep incisions, likely to produce subsequently a great deformity. But in a case where we have to deal with preserving, or even with prolonging, life, we cannot take into account resulting deformity.

On the 21st of January, 1850, the patient having inhaled chloroform, the operation was performed in the same way as in the former case. But the soft parts of the face being destroyed a great deal by this loss of substance, the obtuse-angled flap was separated in two unequal halves.

The maxillo-zygomatic junction was separated at the left side by the chain-saw; at the right by Liston's bone-forceps.

The soft palate having been cut from the bone, the back union between the jaw bone and the pterygoid process of the sphenoid was separated by a chisel introduced through the nose, and employed like a lever. In this way the operation was finished, and both bones could be taken away in pieces of various size. In order to stop the bleeding, the application of ice and the ligature of an artery were quite sufficient.

Immediately after the operation, Professor Heyfelder united only the right cheek wound; then the patient was put to bed, and restored by some broth. Three hours after, a rather important bleeding, from the left incision, took place out of the arteria coronaria, and did not stop until the wound became united.

The loss of substance produced by the resection of the degenerated parts of the nose and lip, Professor Heyfelder tried to cover by autoplasty. Nothing else was resorted to except cold local applications.

The first night passed quietly; the patient awoke refreshed by a long, deep sleep; but his face being swollen, and the parts near the sutures much stretched, the cold fomentations were replaced by tepid epithems of lead wash. The second night passed as well as the first. The fourth day a part of the sutures, on the fifth the rest of them, were removed. The cheek incisions were very well healed, but union in the median line had not succeeded.

The 18th of February, that is to say, the twenty-ninth day after the operation, the ununited parts of the wounds were completely cicatrized. In the inner part of the mouth cicatrization had also taken place. On the upper side of the perpendicular plate of the ethmoid bone, in the soft palate, a round aperture was to be seen. In consequence of the defect in the nose and lip, there was a narrow triangular fissure in the right half of the face, the base of which corresponded with the mouth, and the top spread to the root of the nose.

He was able to drink and eat soft nourishment pretty well; his speech was very unintelligible, but it improved a great deal as soon as the aperture of the palate, and more even when the outer fissure were closed. With this patient, the same as with the former, we made the observation, that the soft parts of the face, though deprived of their essential support by the removal of both upper jaw bones, did not collapse, and that in this way no deformity resulted. On the contrary, after a resection of one jaw bone, the collapse of the skin is not to be avoided, as Prof. Heyfelder's* nine cases, and those of others, have proved.

The microscopical examination of the tumor confirmed it to be cancerous.

From official communications we knew that the man had remained well during twenty months, but then the cancer reappeared, extended itself over the whole face, the eyes, the pharynx and larynx, and he died on the 9th of December, 1851.

Case III.—Johann Baptist Hieroth, aged 21, very thin, of a delicate constitution, but never seriously ill, suffered in the beginning of February, 1852, with vehement pains in the two last back teeth of the left upper jaw bone, which became loose and were extracted. Neverthless, the pain continued, matter came out of the two alveoli, and the alveolar process was ex-

*Amp. and Resect, pp. 29–57.

foliated at this place. Three months later the hard and soft palate swelled, the man spoke through his nose; the pain increased to an intolerable degree. On the 28th of June, 1852, the patient entered our Clinique.

The hard palate was perforated in three places: in the middle, where the perforation was four lines in diameter; on the left side, where the two removed teeth had been; and on the right side, near the second upper back tooth. Out of these three openings flowed a nasty, discolored, ichorous fluid; the probe introduced through these apertures touched granulating masses. The soft parts of the palate were loosened, ulcerating, and of an unhealthy aspect. An ulceration of the soft palate had destroyed almost all the uvula. This ulceration showed, in its form, secretion and edges quite the nature of a cancer, which was confirmed by microscopical examination.

Although there was no mark on his body, nor did his assertions allow us to think that he had suffered from a primary syphilitic affection, neverthless, we made a cautious attempt to cure him with the decoctum zittmanni.* The disease, however, did not improve; but, on the contrary, made rapid progress, so that after a fortnight, the uvula was already lost, and the speech became quite unintelligible.

The malignity of the disease, its rapid progress over the surface, and in the deep tissues, the increasing waste and weakness of the patient, necessitated a rapid limitation of the disease, and justified the resection of both upper jaw bones, which operation alone would suffice to remove all diseased parts. Perhaps the removal of the left upper jaw bone, and the resection of a

* [Decoctum Zittmanni, *Prussian Pharmacopœia*, (Compound Decoction of Sarsaparilla.) "Take of sarsaparilla, sliced, a pound; digest for a quarter of an hour, and then boil for half an hour, in twenty-four pounds of water, having previously added an ounce and a half of saccharated alum, (equal parts of alum and sugar,) half an ounce of calomel, and a drachm of prepared cinnabar, enclosed in a bag; towards the end of the boiling mix in three ounces of senna leaves, an ounce and a half of liquorice root, and half an ounce each of the seeds of anise and of fennel. Filter the decoction to sixteen pounds, allow it to stand for some time to deposit a sediment, and decant the clear liquid."—Ed.]

third of the right upper jaw bone, would have been sufficient, but experience proves that partial resections are very apt to be followed by early relapses. Professor Heyfelder, therefore, undertook, on the 13th of August, the resection of both ossa maxillaria superiora, after the same preparation as in the former cases. His proceeding was the same as in case I, with the exception that the union of both bones with the frontal and nasal bones was divided in one act. The needle conducting the chain-saw was introduced through the inner partitions of the orbital and nasal processes; the partition wall of the nose, the vomer, and the junction with the ethmoid bone, were sawed through. The soft palate being pared from the hard one, the operàtor, with the help of a chisel, tried to enucleate the bones, as in the former cases he had easily succeeded in doing. But this time it could not be effected till the union between both upper jaw bones was separated with the chain-saw. Then the left one broke under the movements of the chisel, and could easily be taken away. Now we had room enough to separate, by means of Liston's bone forceps, the union of the right upper jaw bone with the palate bone, and the pterygoid process of the sphenoid bone, which, in this case, had been extraordinarily strong.

After the ligature of one artery, the bleeding was stopped by the application of the actual caustic, as neither ice nor the liquor Pagliari* had sufficed to stop it.

In consequence of after-bleeding, the wounds were not united until four hours later, and cold fomentations were applied. No remarkable reaction took place, not even swelling of the face. During the first day the man received only broth, by means, first, of a syringe, then of a spoon. The fourth day, the ligatures being removed, the wounds were healed. The 25th of August, when dismissed home, he could eat almost every thing, his speech was more intelligible, the face very slightly deformed. A year later we received from the government physician of his county the information that Hieroth was quite well, without any trace of disease within his mouth; the

*[See Dublin Quarterly Journal, N. S., vol. xiv, p. 502.—Ed.]

hollow produced by the operation being filled up with cicatrice tissue; that he seemed well nourished; that his speech had very much improved; and that he was occupied as a workman on a railway. Meantime, his face was so little altered, and his appearance so good, that nobody would have thought him to have undergone such a severe operation.

Dieffenbach,* in 1848, performed a similar operation, in consequence of a large tumor of the face. He incised the soft parts in the median line, and removed the greater part of both upper jaw bones, together with the palatine bones, and a part of the cheek bone.

Maisonneuve,† in 1849, totally removed both ossa maxillaria superiora, following the same method as Dieffenbach as regarded the cutaneous incision. The operation was for a case of cancer; the man operated on died a few days after.

Maisonneuve,‡ in 1850, performed the same operation upon a girl, who had long been employed in a phosphorus match factory; the subcutaneous extraction of both the upper jaw bones, mortified by necrosis.

Similar operations have been performed by Dietz and Jüngken;§ the latter separated both bones in their median line with the bone-forceps of Liston. Both operations had good success.

Langenbeck,|| in 1853, in case of a medullary carcinoma, removed both upper jaw bones, excepting only the orbital plate of the left one. He divided the skin on the right side, by one oblique incision through the cheek; on the left by a combined incision in this form Λ. The bones were separated by means of a metacarpal saw, a bone forceps, and a chisel.

*The indications for the removal of both upper jaw bones* result from the same diseases as those for the resection of one; only they must be extended over both bones.

*Necrosis and caries* of both upper jaw bones, where no signs

* Operative Chirurgie, t. ii, 46.

† Gaz. des Hôp., 1850, Nos. 97 and 100, and Oscar Heyfelder, Klinischer Bericht in der Prager Vierteljahshr, bd. xxi.

‡ Gaz. des Hôp., 1850, p. 510.

§ Deutsche Klinik, 1850, p. 48.

|| Deutsche Klinik, 1853, p. 204.

of a tendency to cure are visible, but where the whole state of health, or even life, is endangered, require an operation, and, if extended over the greater part of the upper jaw, *the resection of both bones.*

*Benign tumors,* whether they originate from the maxillary sinus, or from the substance of the bone, may, by excessive increase, produce deformity, derangement of the physiological functions, and even danger to life; and, therefore, their removal may be necessary. They require the resection of both upper jaw bones if the entire or the greater part of the bones are diseased, or connected with the tumor; or if the tumor itself cannot be removed without extirpating the bones. *Enchondroma* and *osteosarcoma* give the most frequent occasion for this operation.

*Malignant tumors,* occupying both bones, permit only a partial resection, if limited to the free edges of the bones, for instance, of the alveolar margin. As partial resections of the upper jaw in case of more extended malignant tumors prove to be generally followed by early relapses, the total removal of the bones is, in this case, to be preferred to a partial operation. All the different kinds and forms of cancer have been observed to occupy the upper jaw, and have demanded the extirpation of one or both bones: the *epithelial cancer,** (the cancroid of Virchow,) the *cancer gelatiniforme,*† the *carcinoma medullare,*‡ the *cystocarcinoma.*§

The *contra-indications* are: 1. An excessive extent of the disease, so as not to be totally removed, especially if the base of the skull is equally affected, and if the disease is of a malignant character. On the contrary, superficial connection of benign tumors with the periosteum of the base of the skull does not contra-indicate the operation, as is proved by experience. 2. Cancerous deposition in other organs. 3. Dyscrasy being connected with the local disease, as scrofula, syphilis, &c.

* Michon, Gaz. des Hôp., 1853, p. 178; and Solé, Gaz. des Hôp., 1854, p. 46

† Maisonneuve, Gaz. des Hôp., 1852, p. 139.

‡ Heyfelder, Resect. u. Amp. pp. 30, 33, 34, 37, 50, 57—67.

§ Heyfelder, ibid, p. 42; and Klinischer Bericht, 1853—54, p. 21.

*The Operation.*—The proceeding is analogous to the extirpation of one upper jaw bone, and not at all more difficult than this, the separation of the median junction being unnecessary in the greater number of cases. It consists, essentially, of two acts. The first is the denudation of the bone from the soft parts; the second, the excision of the bones from their osseous connection.

Act I. *Denudation of the Bone.*—The soft parts may be cut by simple or complicated methods. The simple are more used, and, if possible, to be preferred to the complicated:

1. *The median incision*, followed by Dieffenbach and Maisonneuve, commences from the root of the nose, and ends in the middle of the upper lip. If necessary, a second incision from the beginning of the first to the inner corners of both eyes, must be added. In this way we obtain two lateral flaps, which are pared closely from the bones, and held on either side with blunt hooks. By this proceeding the fewest vessels, nerves, and muscles are cut, and the cicatrix produces but little deformity, although it affords room enough to perform the operation.

2. *The posterior lateral incision, or cheek incision*, employed by J. F. Heyfelder in his three cases, begins on each side from the outer corners of the eyes, and finishes at the angles of the mouth; or, to avoid the ductus Stenonianus in the upper lip, some lines within the corner. By this incision, a large flap is obtained, with the base above. This gives more room than any other of the simple methods, but it has the disadvantage of exposing the facial nerves, the principal vessels of the cheek, the ductus Stenonianus, and a great number of muscles, to the knife.

3. *The anterior lateral method* would consist in two incisions beginning at the inner corner of the eye, and descending near the wing of the nose to the upper lip. Hitherto it has not been practiced upon the living, except on one side. But, from my experiments on the dead body, it would appear to be well adapted for both sides. This and the former methods may be usefully combined on the different sides.

In the rare case that these simple proceedings would not suffice to denude excessively enlarged bones, the complicated method must be employed on one or both sides; it was practiced by Malgaigne, Syme, Jaeger, Lisfranc, Blandin, Fergusson, and others, for the resection of one upper jaw bone.

Instead of the crucial and flap incisions, which always expose the soft parts a great deal, and produce much deformity, J. F. Heyfelder proposes for those cases, where the simple methods do not suffice, the following proceeding: One incision slits the under lip in the middle, and proceeds to the chin; from thence a second and third incision extend themselves on both sides along the under edge of the mandibule, and the hind edge of the ramus ascendens. By this method the nerves and Steno's duct are preserved, and the cicatrix falls where it may easily be concealed—in men, by a beard, in women, by a handkerchief.

The soft parts being cut through, they are pared from the bones, preserving, if possible, the periosteum on that side, and the flaps thus formed are held back by an assistant.

Act II. *The Excision of the Bones.*—We commence with the separation of the maxillo-zygomatic juncture on either side, which is best effected by the chain-saw. The introduction of the needle and chain-saw in the inferior orbital fissure, and the advance of the instruments behind the zygomatic bone, is imagined as the most difficult part of the operation; and some German and French authors were inclined to ascribe this difficulty to the narrowness of the fissure, (Ried, Heyfelder, Chassaignac, Lisfranc, Roux, De Brignolles.) But after measuring sixty fissures, I have found that their anterior opening is usually widened (*sinus fissuræ orb. inf.*,) and shows a medium breadth of $2\frac{1}{3}$ lines, which affords space enough to introduce an ordinary needle. Even in case of disease of the upper jaw, the bony edges forming the inferior orbital fissure, if attacked by the disease, are oftener destroyed and thinned by pressure, osteoporosis, or caries, than thickened and covered with osteophyts.

The real difficulty arises from two different reasons: 1. Operators usually choose needles too little curved, so that the point cannot protrude below the cheek bone, as the head of the needle is approached to the inferior orbital margin; or their needle is provided with too sharp a point, which often engages itself in the periosteum, and even in the substance of the bones. 2. The needle, which is held but very loosely by the operator, is apt to turn between the fingers and to follow a false direction; so that he is often obliged to withdraw and reintroduce it several times.

To avoid this difficulty, I use a very curved needle, forming about two-thirds of a circle, with $\frac{7}{8}$ in. semidiameter, having no sharp point, and introduce it by means of a pincers, constructed for this purpose. The extreme ends of the pincers have, on the inside, a little groove corresponding to the four-cornered head of the needle, so that it can be fixed in the ends of the pincers, and thus be introduced in the direction intended. By means of these very simple instruments, the introduction of the chain-saw behind the maxillo-zygomatic junction has always succeeded with remarkable facility.

The separation of the upper and posterior connections is the same as with the resection of one upper jaw bone; only the two upper jaw bones may be disjointed from the frontal and nasal bones by one act, according to a proposition of Maisonneuve, executed by J. F. Heyfelder in his fifth case.

The vomer, as well as the union of the maxillary bone with the cribriform bone, is cut with a bone-forceps. At this part of the operation the soft palate is cut from the hard one by a small knife, the blade being conducted in a flat manner.

The dressing and after-treatment do not differ from the same after the resection of one upper jaw bone. Our method of not closing the wound until some hours after the operation, to avoid the necessity of opening it again in case of after-bleeding, is always successful. The union was effected by the suture nodoso, or circumvoluta, or by *serres-fines;* the latter can only be employed upon parts which have no tension.

The success of the operation must be called good. The wound in the soft parts usually heals by the first intention, within four or five days. It is astonishing that even with this extirpation the loss of substance is almost completely filled up by granulations, and any openings that may remain can be closed with an obturator. Speech becomes by-and-by more intelligible; the senses of seeing, hearing, smelling—sometimes lost from the pressure of the tumor—return, sooner or later, after the operation; the faculty of eating even substantial meats is re-acquired; and the deformity is very slight.

Of nine that were operated on, four died; in one case the final result is unknown; in four it was successful. One of the patients seems to have succumbed to the operation, that is to say, the first operated on by Maisonneuve; one died from an apopletic seizure—that was the man operated on by Dieffenbach; two (Nos. 1 and 2 of those operated on by Heyfelder) succombed to a relapse of cancer, but one not until fifteen, the other twenty-three, months after the operation; which relapses appearing after such a length of time, the result cannot be called otherwise than successful.

*Literature of the Subject.*

J. F. Heyfelder: Klinischer Bericht von 1843–44, in Walthers und von Ammon's Journal, B. iv. Heft 4, 1844.

Idem: Ueber die Resektion beider Oberkiefes, Stuttgart, 1850.

Idem: Klinischer Bericht, von 1851–52. Deutsche Klinik, 1852.

Idem: Amputationen und Resectionen. Breslau, 1854.

Ried: Die Resektionen der Knochen, 1847.

Dieffenbach: Operative Chirurgie, 1848.

Maisonneuve: Gaz. des Hôp., 1850. Nos. 97, 100, und 510.

Oscar Heyfelder: Klinischer Bericht von 1849–50, in der Prag. Vierteljahrschrift, Bd. xxi.

Idem: Artikel: Resectionen in Prosch Handwörterbuch. Leipzig, 1854.

Malgaigne: Revue Médico-chir. Tome xiii.

Jüngken: Deutsche Klinik, 1850, p. 48.

Langenbeck: Deutsche Klinik, 1853, p. 204.

[*Dublin Quarterly Jour. Med. Sci.*

